# Supplementary material for: Differential Effects of Endocannabinoids on Amyloid-Beta Aggregation and Toxicity
Source: Int J Mol Sci. 2023 Jan 4;24(2):911. doi: 10.3390/ijms24020911 (PMC9861930; doi:10.3390/ijms24020911)
Supplement: Supplementary file 1 [file ijms-24-00911-s001.zip › ijms-2104821-supplementary.pdf]

Supplementary Information

Differential Effects of Endocannabinoids on Amyloid-Beta Aggregation and Toxicity

Marzie Khavandi, Praveen P. N. Rao and Michael A. Beazely *

School of Pharmacy, Health Sciences Campus, University of Waterloo, Waterloo, ON N2L 3G1, Canada; marzi.khavandi@uwaterloo.ca (M.K.); praveen.nekkar@uwaterloo.ca (P.P.N.R.)
* Correspondence: mike.beazely@uwaterloo.ca; Tel.: +(1)-519-888-4567 (ext. 21310)

Table S1

Compd Concentration	2AG	AEA	NADA	Noladin	OAE	AA
10 µM	12.8±00	93.5±1%	18.7±6.4%	72.9±15%	25.1±1.2%	94.5±0.6%
5 µM	3.5±1%	14.5±4.3%	8.3±4.9%	14.6±6.8%	11±6.1%	86±5.3%
1 µM	22.8±1.9%	29.0±8.3%	37±13.1%	19±9.2%	14.7±2.3%	25.3±7.5%

Table S1. The inhibition percentage of ThT-monitored aggregation kinetics of Aβ42 (5 µM) in the presence of 1, 5, 10 µM of endocannabinoids at pH 7.4, 37 °C in phosphate buffer at 24 h. Aggregation kinetics were monitored by ThT fluorescence spectroscopy (excitation = 440 nm, emission = 490 nm). Results are average ± SD of three technical replicates.

Figure S1

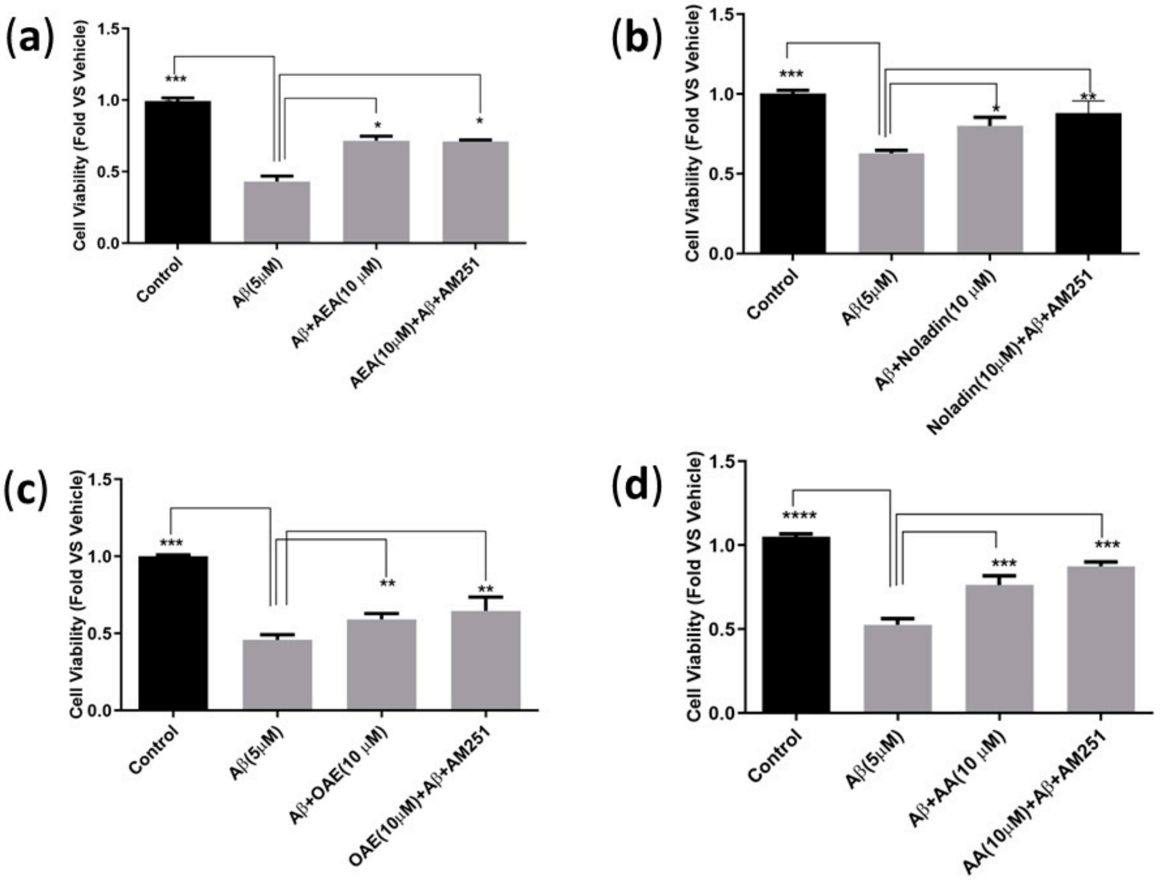

Figure S1. Effect of at 1, 5, 10 μM endocannabinoids and AA, against Aβ42 (5 μM) toxicity on HT22 cells. Cells were treated with Aβ42 in the absence or presence of AEA (A), Noladin (B), OAE (C),and AA (D). The CB1 receptor antagonist, AM251 (5 μM), was also used. Cell viability was determined via MTT assay and data are expressed as fold change in viability vs. control. A one-way ANOVA with Dunnett's multiple comparisons performed to establish significance between groups (α = 0.05*). The results are shown as the average ± standard error of the mean (SEM). The data is representative 4 independent experiments, each with 4 technical replicates.

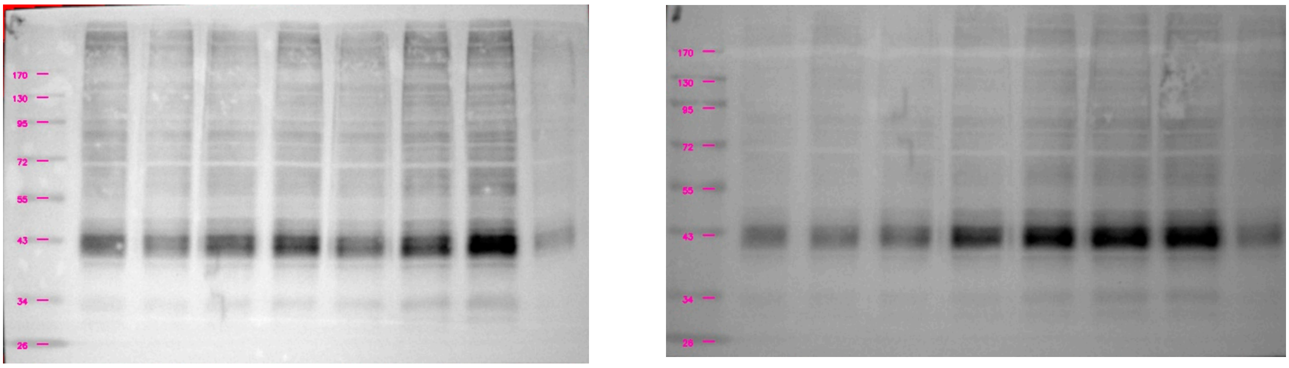

Figure S2. CB1-CHO cells were lysed and lysates evaluated by Western blots as described in the methods. We detected the presence of the CB1 receptor band close to the expected molecular mass of the CB1 receptor (approximately 43 kDa). The lanes are different concentrations of cells from 5μg to 20μg. The experiment was performed in duplicate and both blots are shown.
